# Changes in the blood redox balance during a simulated duathlon race and its relationship with athletic performance

**DOI:** 10.14814/phy2.14277

**Published:** 2019-11-06

**Authors:** Takamasa Tsuzuki, Kei Tsukioka, Hisashi Naito

**Affiliations:** ^1^ Faculty of Pharmacy Meijo University Nagoya Japan; ^2^ Graduate School of Health and Sports Science Juntendo University Inzai Japan

**Keywords:** Duathlon, oxidative stress, performance, simulated race

## Abstract

The duathlon is an endurance multisport event that consists of sequential running, cycling, and further running. Imbalance in the redox homeostasis is associated with fatigued status and underperformance in various sports; however, there are no corresponding reports regarding the duathlon. The purpose of this study was to examine the changes in the blood redox balance during a simulated duathlon race and to determine the relationship between performance and the redox balance. Eight male triathletes participated in a simulated race, consisting of a 5‐km run, 30 km cycling, and a further 5‐km run, with 5 min rest between two parts to collect the blood samples. The serum levels of reactive oxygen metabolites (d‐ROMs) and biological antioxidant potential (BAP) were measured and BAP/d‐ROMs ratio and oxidative stress index (OSI) were calculated. The d‐ROMs levels after the first Run were significantly increased compared with the levels observed before the race. Moreover, BAP levels increased significantly over the race. The BAP/d‐ROMs ratio also gradually increased through the race, while the OSI was gradually decreased. In addition, a significant relationship was observed only between d‐ROMs levels after the first Run and the first Run performance. These results suggest that the redox balance shifts toward reduction (antioxidation) during the duathlon race and increased oxidant potential levels are negatively correlated with performance in the early stages of the race.

## Introduction

The duathlon is an endurance multisport event that consists of sequential running (1st Run), cycling, and further running (2nd Run). This event is internationally governed by the International Triathlon Union (ITU). The most common configuration for a duathlon race is a 5‐km run, 30 km cycling, and a further 5 km run (Sparks et al. 2005).

Generally, aerobic exercise increases the production of reactive oxygen species (ROS), resulting in oxidative stress and/or imbalance of redox homeostasis (Nikolaidis et al. 2012). ROS production is a fundamental feature of mammalian physiology, cellular respiration, and cell signaling, and is essential for muscle function and training adaptation (Powers et al. 2011; Merry and Ristow 2016; Reid 2016). However, an overwhelming increase in ROS may lead to increased cell apoptosis and immunosuppression, a fatigued state, and underperformance (Lewis et al. 2015). Thus, it is important for athletes to evaluate and understand their redox balance to maintain their physical condition and ensure good athletic performance.

Several previous studies reported that levels of oxidative markers such as malondialdehyde (MDA), oxidized glutathione (GSSG), and 8‐OHdG increased after competing in sports such as marathons (Gomez‐Cabrera et al. 2006; Withee et al. 2017), cycling (Almar et al. 2002; Aguilo et al. 2005) and triathlons (Knez et al. 2007), and the increase in oxidative marker levels was negatively correlated with exercise performance (Lewis et al. 2015); however, these assay methods are complex and are not often stable enough for use in the sports science field. In addition, there are no similar reports regarding the duathlon. In the case of multiactivity sports like the duathlon and triathlon, the data regarding the alterations in redox balance during a race, not just after the race, are extremely valuable because exercise types (i.e. swimming, cycling, and running) are changed during the race.

In the field of sports science, useful and noninvasive biomarkers are required to measure the redox balance anytime and anywhere (*e.g.* outside the athletic field). As simpler evaluation markers of redox balance, reactive oxygen metabolites (d‐ROMs) and biological antioxidant potential (BAP) tests were developed (Alberti et al. 2000); they offer convenience and rapid results. The d‐ROMs test provides a measure of the whole oxidant potential of plasma against *N,N*‐diethyl‐para‐phenylenediamine, which is mainly due to hydroperoxides with the contribution of other minor oxidant factors (Alberti et al. 2000; Trotti et al. 2002). On the other hand, the BAP test enables evaluation of the ferric reducing ability of plasma as antioxidant potential, which is attributed to the major component of the plasma barrier to oxidation (vitamin C, vitamin E, uric acid, bilirubin, and so on) (Benzie and Strain 1996; Dohi et al. 2005; Hetyey et al. 2007). They provide prompt feedback to athletes and are suitable to be used as in the sports field testing tools.

Therefore, the aim of the present study was to examine the changes in the blood redox balance during a simulated duathlon race using the d‐ROMs and BAP tests and to determine the relationship between the redox balance status and athletic performance.

## Methods

### Subjects

Eight male college triathletes participated in this study. The characteristics of the subjects are shown in Table 1. All subjects were free of overt chronic diseases according to their medical history. They were informed of the purpose, procedure, and risks of the study and signed an informed consent form before participating in the study. This study conducted in accordance with the principles of the Declaration of Helsinki and was approved by the Human Ethics Committee of Juntendo University, Faculty of Health and Sports Science (29‐10).

**Table 1:** Characteristics of the subjects.

Age (year)	21.0 ± 1.5
Height (cm)	170.8 ± 1.4
Weight (kg)	64.4 ± 5.3
BMI (kg·m^−2^)	22.1 ± 1.7
Body fat (%)	12.9 ± 1.9

The time in total and each part were recorded as the performance. The data are expressed as mean ± standard deviation.

### Study design

**Figure 1:**
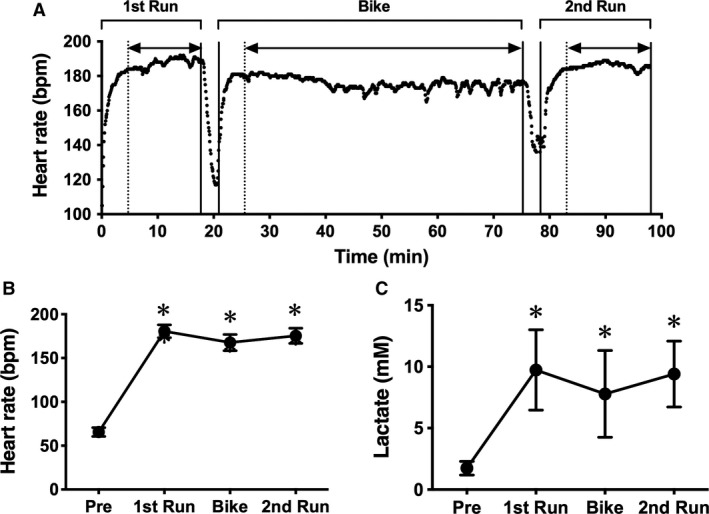
Heart rate (HR) and lactate concentration during a simulated duathlon race. A typical example of time course changes in HR during the race (A) and averaged HR in each part (B) is shown. Double‐headed arrow indicates the time span used to calculate the average HR. Lactate concentration (C) was measured before the race and after each part. The data are expressed as mean ± SD. *P < 0.05 versus pre.

To examine the changes in oxidative stress markers during the duathlon, all subjects were participated in a simulated duathlon race that consisted of a 5‐km first Run (first Run), 30 km cycling (Bike) and 5‐km second Run (second Run). On the day before the race day, the participants fasted after 9 p.m. and had refrained from exercise for at least 24 h before the race. On the morning of the race day, the height, weight, and body composition of the subjects were measured using an InBody730 device (InBody, Tokyo, Japan). Small amounts of blood samples (~100 *μ*L) were taken from the participants’ fingertips before the race and immediately after the first Run, Bike, and second Run. The race time in each part was recorded as the index of performance. Heart rate (HR) was monitored during the race using a Polar RS800CX device (Polar Electro In., Finland); the average HR in each part, except for the initial 5 min, was calculated using Polar Protrainer software (Fig. 1A). The subjects were allowed to drink water ad libitum during the race.

### Measurements of blood markers

The lactate concentration was measured using the Lactate Pro device (Arkray, Kyoto, Japan). In addition, the blood samples were centrifuged at 800 *g* for 2 min to collect serum samples. The serum levels of d‐ROMs and BAP were measured using a FREE Carrio Duo (Diacron International, Grosseto, Italy) according to manufacturer’s instructions. Briefly, in the d‐ROMs test, hydroperoxides are converted into radicals that oxidize *N,N*‐diethyl‐para‐phenylenediamine and can be detected spectrophotometrically using an all‐purpose automatic analyzer. The results of the d‐ROMs analysis were expressed in Carratelli units (U. CARR.), where 1 U. CARR. is equivalent to 0.08 mg/dL H_2_O_2_ (Trotti et al. 2002; Pasquini et al. 2008). The BAP level is measured to determine the antioxidant potential. This test examines the blood concentration of antioxidants as agents that can reduce iron from the ferric (Fe^3+^) to the ferrous form (Fe^2+^). The results are expressed in μmol/L of the reduced ferric ions (Benzie and Strain 1996; Pasquini et al. 2008). In our laboratory, the intraassay coefficients of variation (CV) for our device ranged from 0.5 to 2.5% for d‐ROMs and from 0.9 to 2.2% for BAP, respectively. A previous study also reported similar range of the CV (d‐ROMs: 0.2 − 2.1%, BAP: 0.1–1.1%) (Fukui et al. 2011). Moreover, the validity of d‐ROMs test has been demonstrated by comparisons with the results of the electron spin resonance (ESR) method (Alberti et al. 2000), and both tests showed highly linear and accurate, as previously described (Benzie and Strain 1996; Iamele et al. 2002; Trotti et al. 2002; Pasquini et al. 2008). To obtain a parameter representing an overall shift toward the oxidative stress, the oxidative stress index (OSI) was devised using the following formula: OSI = *C* × (d‐ROMs/BAP), where *C* denotes a coefficient for standardization to set the mean of OSI in Pre at 1.0 (*C* = 8.11 in this study) (Nojima et al. 2011).

### Statistical analysis

Data are expressed as mean ± standard deviation (SD). Statistical significance was determined by using the repeated measures ANOVA followed by a Bonferroni’s multi comparisons test. Correlations between redox markers and race performance were analyzed using simple linear regression and Pearson’s correlation analysis. *P* < 0.05 was considered statistically significant. All statistical analyses were performed using PRISM v.8.0.1 software (GraphPad software, San Diego, CA).

## Results

### Performance in the duathlon race

The simulated duathlon race conducted in this study consisted of a 5‐km first Run, 30 km cycling, and a 5‐km second Run. All subjects completed the competition. The average time required for the race is shown in Table 2.

**Table 2:** Results of a simulated duathlon race.

Race time	
Total	1° 34' 29" ± 6' 08"
First Run	18' 36" ± 1' 56"
Bike	56' 18" ± 3' 41"
Second Run	19' 35" ± 1' 46"

The time in total and each part were recorded as the performance. The data are expressed as mean ± standard deviation.

### Exercise intensity during the duathlon race

We monitored the subjects’ HR during the simulated race. A typical example of monitored HR during the simulated duathlon race is shown in Fig. 1A. The average HR in the first Run, Bike, and second Run was 181 ± 7, 170 ± 11, and 177 ± 9 bpm, respectively (Fig. 1B). These values were found to be 90.8 ± 3.4, 85.4 ± 5.1, and 88.8 ± 4.5% of the maximum HR speculated regarding age, respectively. In addition, lactate concentrations after each part were 9.7 ± 3.3, 7.8 ± 3.5, and 9.4 ± 2.7 mmol/L, respectively (Fig. 1C). These data suggested that the race was carried out at very high intensity.

### Changes in oxidative stress markers during the duathlon race

**Figure 2:**
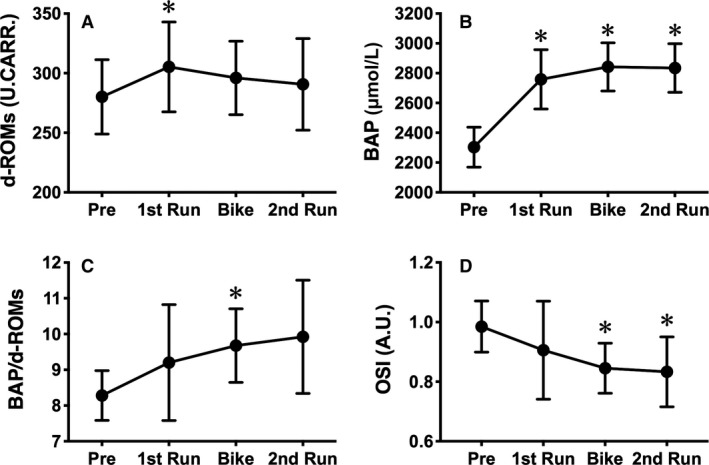
Changes in redox markers during a simulated duathlon race. The levels in d‐ROMs (A) and BAP (B) were measured before the race and after each part. BAP/d‐ROMs ratio (C) and OSI (D) were calculated with the values of d‐ROMs and BAP in each point. The data are expressed as mean ± SD. *P < 0.05 versus pre.

To reveal the changes in redox balance during the duathlon race, we measured d‐ROMs and BAP levels after each part. d‐ROMs levels after the first Run were significantly increased compared with the levels observed before the race and were slightly decreased afterwards (Fig. 2A). On the other hand, BAP levels after each part were higher than that before the race (Fig. 2B). Next, to assess the redox balance during the race, BAP/d‐ROMs ratio and OSI were calculated. Similar with BAP levels, the BAP/d‐ROMs ratio gradually increased through the race and were significantly higher after the Bike part than before the race (Fig. 2C). Conversely, OSI was gradually decreased and was significantly lower after the Bike and second Run parts than before the race (Fig. 2D).

### Relationship between performance and redox markers

**Figure 3:**
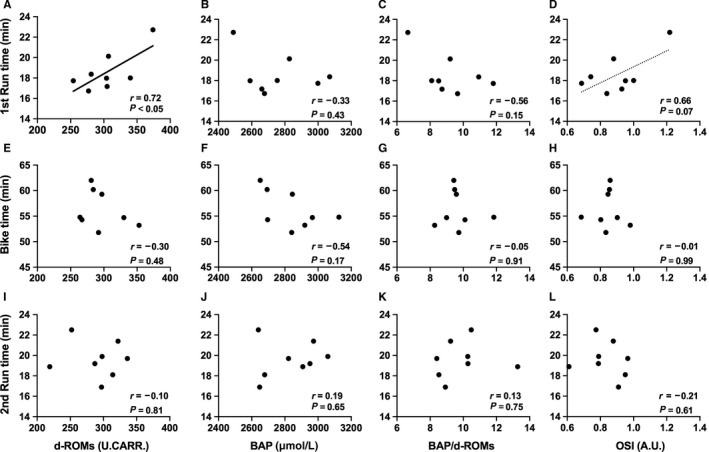
Correlation between race time and redox markers after each part. The correlations between d‐ROMs, BAP, BAP/d‐ROMs, OSI after each part and race time in first Run (A–D), Bike (E–H), second Run (I–L) parts were shown. The regression line was described when a *P*‐value was smaller than 0.05.

There were no significant relationships between redox markers before the race and total and each part of race time (data not shown). On the other hand, a significant relationship was observed only between the d‐ROMs levels after the first Run and race time required for first Run part (*r* = 0.72, *P* < 0.05), suggesting that higher levels of oxidant potential were negatively related to running performance in first Run part (Fig. 3).

## Discussion

In the present study, we examined the changes in redox balance during a simulated duathlon race and the relationship between the redox markers and performance. We found that d‐ROMs and BAP were significantly increased in different phases during the race, with the ratio of the two measures indicating an overall reduction in oxidative stress through the duathlon race. Furthermore, we identified a significant relationship between the d‐ROMs level after the first Run and the first Run performance. Thus, our data partially support the concept that redox balance influences the exercise performance. To our knowledge, this is the first study to evaluate blood redox balance during a duathlon race and its relationship with performance.

We observed that the d‐ROMs levels were higher only after the first Run than before the race; the levels decreased slightly afterwards. It is thought that this elevation in d‐ROMs is largely due to the increased production of ROS in the initial phase of exercise through a rapid increase in oxygen demand, followed by temporary hypoxia in the cells due to insufficient oxygen supply (Smith et al. 2017) as well as increased the blood flow (Durand and Gutterman 2014). Subsequently, a rise in the d‐ROMs in the latter stage of the race might not be observed because the scavenging ROS are prompted by the increase in antioxidant capacity, as seen based on the BAP levels. BAP levels are an indicator of total antioxidant capacity and are influenced by several factors like the presence of endogenous enzymatic and nonenzymatic antioxidants (Finaud et al. 2006). Indeed, some previous studies have reported that the activity of antioxidant enzymes such as superoxide dismutase, catalase, and glutathione peroxidase increases in the blood and in tissues after endurance exercise (Ji 1993; Clarkson 1995). It has also been shown that endurance exercise causes an increase in the serum levels of antioxidants such as vitamin A, C, and E (Wiecek et al. 2015), uric acid (Liu et al. 1999), melatonin (Atkinson et al. 2003), and bilirubin (Benitez et al. 2002). Thus, the rise in BAP levels during the race may be partially accounted for the increase in these parameters; however, these were not tested in the current study. To reveal the changes in redox balance during the race, we used only the BAP/d‐ROMs ratio and OSI. These data suggest that the increase in BAP was greater than the increase in d‐ROMs, indicating that the redox balance shifted toward reduction (antioxidant). Similar to our results, a study by Lewis et al. (2016) showed that the OSI, which is calculated based on other redox parameters, declined in response to submaximal and maximal exercise in male and female athletes. Further study is needed to determine if these indicators are useful for determining athletes' conditioning and assessing their performance.

Some previous studies have reported the relationship between oxidative stress and exercise performance regarding several sports (Knez et al. 2007; 2014); however, to the best of our knowledge, there are no reports of this nature regarding the duathlon. In the simulated duathlon race we conducted, the results showed that the increase in d‐ROMs after the first Run is correlated with the performance in the first Run. Therefore, this data partially support the opinion that oxidative stress impairs exercise performance. However, this relationship was not observed in the latter stage of the race. As described above, the increase in BAP was maintained through the race and it is possible that endogenous antioxidants can scavenge enough of the ROS generated by exercise in the latter stage. Moreover, Margaritis et al. (1997) showed that the level of the reductive form of glutathione before a triathlon race was significantly correlated with race performance. Taken together, enhancing antioxidative capacity (e.g. antioxidant supplementation) before a competition might contribute to a good performance in the early stages of the competition. However, a previous study reported that ingestion of antioxidants prior to exercise negatively affects some physiological markers and exercise performance (Vidal et al. 2017). Therefore, the effects of antioxidant supplementation on exercise performance remain as a matter to be discussed further.

There are a number of limitations in this study. First, we did not measure the other redox markers except for d‐ROMs and BAP and hemoglobin concentration, hematocrit, and total blood volume to calculate the change in plasma volume (Dill and Costill 1974), because the blood samples were taken in small amounts. However, one of the merits of the d‐ROMs and BAP tests is that the levels can be measured with small blood samples. Therefore, we chose these tests for their high potential for in the field testing with athletes. Moreover, plasma volume is decreased by dehydration during prolong exercise, which in turn affects the concentration of blood biomarkers. Thus, in future studies, it is necessary to confirm the influence of change in plasma volume by dehydration during prolonged exercise on d‐ROMs and BAP levels. Second, we did not measure any index of fitness level such as maximum oxygen consumption or anaerobic threshold. Further studies are needed to determine the relationship between fitness levels and oxidant potential or oxidative stress index measured in this study. Third, we did not control the athletes’ diet before the race. The redox status would be influenced by the antioxidants contained in the daily diet. Since, however, the d‐ROMs and BAP levels before the race were not correlated with the race performance, the effect of daily diet on exercise performance might be negligible in this study.

In conclusion, both oxidative and antioxidative markers are increased in the early stage of the race, thus redox balance is maintained. However, as the increase in antioxidative makers continues until the end of the race, the redox balance shifts toward reduction (antioxidant), suggesting that endogenous antioxidants can scavenge enough of the ROS generated by exercise in the latter stage. In addition, an increase in the oxidant potential level is negatively correlated with race performance in the early stage.

## Conflict of Interest

No conflict of interests, financial or otherwise, is declared by the authors.
